# MicroRNA-101 reverses temozolomide resistance by inhibition of GSK3β in glioblastoma

**DOI:** 10.18632/oncotarget.12861

**Published:** 2016-10-25

**Authors:** Tian Tian, Ma Mingyi, Xia Qiu, Yang Qiu

**Affiliations:** ^1^ Department of Neurology, The First Affiliated Hospital of Zhengzhou University, Zhengzhou 450052, People's Republic of China; ^2^ Department of Neurology, Institute of Clinical Medicine, The First Affiliated Hospital of Zhengzhou University, Zhengzhou 450052, People's Republic of China; ^3^ Department of Medicine, Shangqiu Medical School, Shangqiu 476000, Henan Province, People's Republic of China; ^4^ Department of Clinical Medicine, Shaoyang Medical College, Shaoyang 422000, Hunan Province, People's Republic of China

**Keywords:** glioblastoma, temozolomide, chemoresistance, microRNA, prognosis

## Abstract

Glioblastoma multiforme (GBM) is a chemotherapy-resistant brain tumor with limited treatment options. Temozolomide (TMZ), an alkylating agent, is a front-line chemotherapeutic drug currently employed in GBM. Although it is currently the most promising chemotherapy for GBM, resistance to TMZ is also common and accounts for many treatment failures. Therefore, understanding the underlying mechanisms that generate resistance is essential to develop more effective chemotherapies. Here, we show that microRNA-101 (miR-101) was significantly downregulated in TMZ-resistant GBM cells and human specimens. Instead, over-expression of miR-101 could sensitize resistant GBM cells to TMZ through downregulation of glycogen synthase kinase 3β (GSK3β). Moreover, we found that GSK3β inhibition could enhance TMZ effect through repression of MGMT via promoter methylation. Importantly, decreased expression of miR-101 is related to poor prognosis in patients with GBM, suggesting its potential role as a new prognostic marker in GBM. In conclusion, our study demonstrates that miR-101 can reverse TMZ resistance by inhibition of GSK3β in GBM, thus offer a novel and powerful strategy for GBM therapy.

## INTRODUCTION

Glioblastoma multiforme (GBM), also known as glioblastoma and grade IV astrocytoma, is characterized by a heterogeneous population of cells that are genetically unstable, highly infiltrative, angiogenic, and resistant to chemotherapy [1–3]. Temozolomide (TMZ) is a standard chemotherapeutic drug currently employed in the therapy of GBM [4, 5]. It is an alkylating agent that induces apoptosis through DNA strand breaks [6]. Although chemotherapy with TMZ may enhance survival for patients with GBM, intrinsic or acquired resistance to TMZ is also common and accounts for many treatment failures [7, 8]. Various studies have been conducted to investigate how GBM cells acquire resistance to TMZ [9]; however, the mechanisms are still largely unknown. Therefore, understanding the underlying molecular mechanisms that generate resistance is essential to develop more effective chemotherapies.

MicroRNAs (miRNAs) are a class of small noncoding RNAs proposed to have important roles in the regulation of diverse cellular processes [10]. In recent years, miRNAs have gained significant attention in proliferation, aggressiveness and metastases development of cancer [11]. Increasing evidence has suggested that miRNAs are involved in the development and progression of GBM [12–14]. MicroR-101 (miR-101), which functions as a tumor suppressor, is downregulated in a variety of malignancies including GBM [14]. Studies show that downregulation of miR-101 can induce the proliferation, migration, and angiogenesis of GBM cells [15, 16]. Hence, we speculate that miR-101 may participate in the regulation of TMZ resistance in GBM.

In the present study, we found that miR-101 was significantly downregulated in TMZ- resistant GBM cells, and its re-expression could reverse TMZ resistance through regulation of a serine/threonine protein kinase, glycogen synthase kinase 3β (GSK3β)[17, 18].

## RESULTS

### MicroR-101 is downregulated in TMZ-resistant GBM cells and human specimens

**Figure 1:**
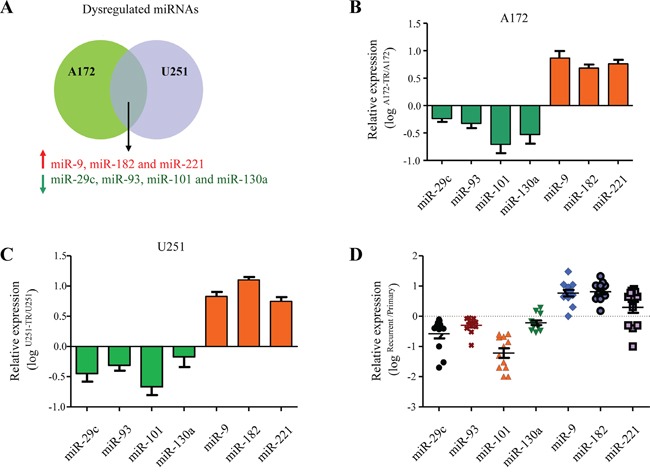
MicroR-101 is downregulated in TMZ-resistant GBM cells and human specimens **A.** Dysregualted miRNAs in TMZ-resistant A172 and U251 cells identified by a miRNA PCR array representing 84 mature miRNAs associated with brain tumorigenesis. Upregulated miRNAs (miR-9, miR-182 and miR-221) were shown in red, downregulated miRNAs (miR-29c, miR-93, miR-101 and miR-130a) were shown in green. **B, C.** The dysregualted miRNAs were confirmed by qRT-PCR in both A172-TR (B) and U251-TR cell lines (C). **D.** The dysregualted miRNAs were confirmed by qRT-PCR in tumor samples obtained from matched pairs of primary and recurrent TMZ-refractory GBM patients (n=12).

To explore the role of miRNAs in the development of TMZ resistance, we first established two TMZ-resistant GBM cell lines using A172 and U251 cells (A172-TR and U251-TR), and then performed a miRNA PCR array representing 84 mature miRNAs associated with brain tumorigenesis to compare miRNA expression changes in TMZ-resistant cells and its parental cells. Seven miRNAs (downregulated: miR-29c, miR-93, miR-101 and miR-130a; upregulated: miR-9, miR-182 and miR-221) were identified as differentially expressed (≥2-fold) in both A172-TR and U251-TR cell lines (Figure 1A). We further validated these results by quantitative reverse transcription-PCR (qRT-PCR) in both A172-TR and U251-TR cell lines (Figure 1B and 1C).

We next sought to determine whether these seven miRNAs were dysregulated in the recurrent TMZ-refractory GBM. To address this question, we examined the expression of these seven miRNAs in tumor samples obtained from matched pairs of primary and recurrent TMZ-refractory GBM from 12 patients. Among them, three miRNAs (miR-29c, miR-93 and miR-101) were downregulated and two (miR-9 and miR-182) were upregulated in the TMZ-refractory samples as compared with that in the primary tumor samples (p<0.05, Figure 1D). The expression of two other miRNAs (miR-130a and miR-221) didn't change significantly (p>0.05). Because of the largest fold change of miR-101 among the three downregulated miRNAs, we chosen miR-101 for the subsequent studies.

### MicroR-101 sensitizes resistant GBM cells to TMZ

**Figure 2:**
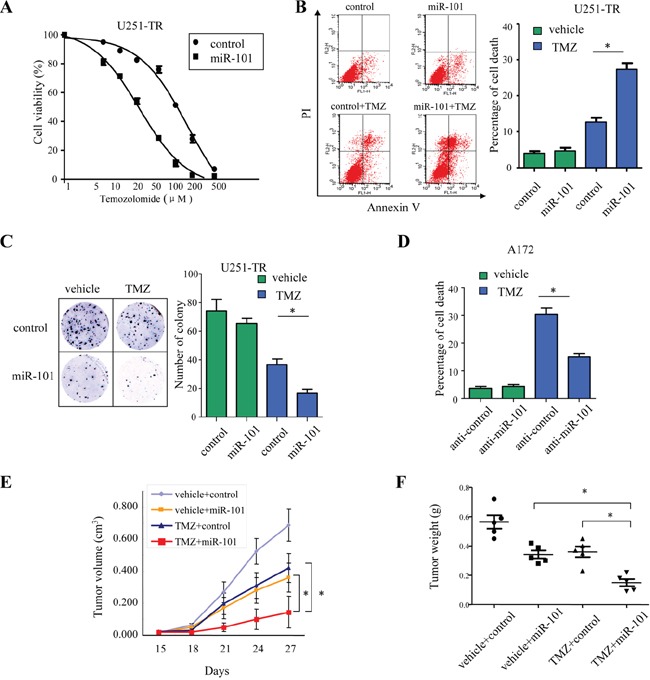
MicroR-101 sensitizes resistant GBM cells to TMZ **A.** TMZ-resistant U251-TR cell lines were transduced with miR-101 and then treated with different concentrations of TMZ for 72 h. The percentage of cell survival over a range of drug concentration was plotted. **B.** The apoptotic rates of U251-TR cells transduced with miR-101 upon treatment with TMZ (200 μM) for 36 h were measured by flow cytometry. *p < 0.05. **C.** Colony formation assay showed the numbers of colonies of U251-TR cells transduced with control or miR-101 in the presence or absence of TMZ (200 μM). *p < 0.05. **D.** The apoptotic rates of A172 cells transfected with anti-miR-101 or control in the presence or absence of TMZ (200 μM) for 36 h were measured by flow cytometry. *p < 0.05. **E.** Tumor size of subcutaneous xenografts measured every three days after two weeks of implantation. The tumor volume was calculated using the formula: (Length × Width^2^)/2. *, *p*<0.05. **F.** The weight of tumor xenografts generated in mice five weeks after inoculation. *, *p*<0.05. All data represent the means ± SEM of three replications.

Since the expression of miR-101 was decreased in both TMZ-resistant GBM cells and human specimens, we next examined whether re-expression of miR-101 could sensitize the resistant GBM cells to TMZ. To address this question, TMZ-resistant U251-TR cell lines were transduced with miR-101 and then treated with different concentrations of TMZ. Results showed that forced miR-101 expression rendered U251-TR cells more sensitive to TMZ. The half maximal inhibitory concentration (IC50) values of U251-TR cells transduced with vector control and miR-101 are (165.0±52.5) μM and (22.4±4.5) μM, respectively (Figure 2A). The flow cytometry assay showed that U251-TR cells transduced with miR-101 exhibited increased cell apoptosis when compared with the vector control group (Figure 2B). We further performed colony formation assay. As shown in Figure 2C, in the absence of TMZ, miR-101 alone could decrease the number of colonies of U251-TR cells. Moreover, miR-101 sensitized U251-TR cells to TMZ treatment, as indicated by significantly decreased number of colony formation (Figure 2C). We also assessed the effect of miR-101 inhibition on TMZ resistance in A172 cells. The results showed that inhibition of miR-101 led to decreased cell apoptosis after TMZ treatment (Figure 2D and Supplementary Figure S3A). It suggested that suppression of miR-101 might render GBM cells resistant to TMZ *in vitro*.

We further evaluated the effect of miR-101 on TMZ resistance *in vivo*. TMZ-resistant U251-TR cells stably transduced with either miR-101 or vector control were injected subcutaneously into two groups of nude mice (n=5). The mice were injected intraperitoneally with TMZ (20 mg/kg/day) every three days after two weeks of implantation. As expected, the tumor growth curve showed that tumors derived from miR-101 group grew more slowly than those from the vector control group. In the absence of TMZ, the tumor volumes of miR-101 group and vector control group were (0.36 ± 0.07) cm^3^ and (0.68 ± 0.10) cm^3^, respectively (*, p<0.05; Figure 2E). Upon TMZ treatment, the tumor volumes of miR-101 group and vector control group were (0.14 ± 0.09) cm^3^ and (0.42 ± 0.08) cm^3^, respectively (*, p<0.05; Figure 2E). Moreover, in the presence of TMZ, the mean tumor weight was significantly lower in the miR-101 group (0.15 ± 0.02 g) compared to that of the vector control group (0.36 ±0.04 g; *, p<0.05; Figure 2F and Supplementary Figure S3B). These results indicated that miR-101 sensitized resistant GBM cells to TMZ *in vivo*.

### GSK3β is a direct target of miR-101

**Figure 3:**
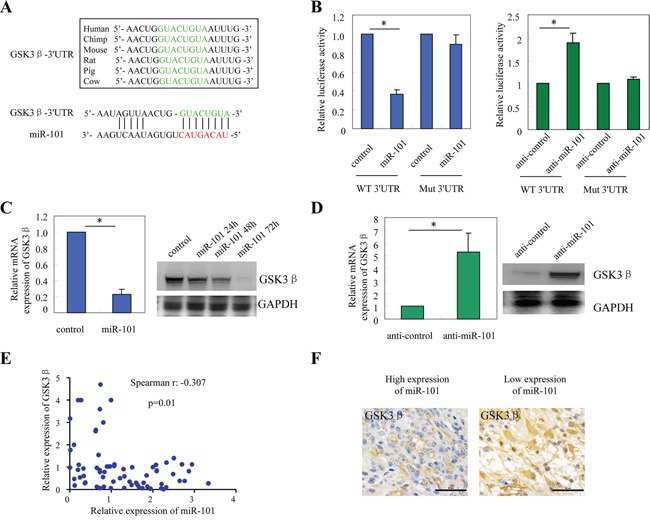
GSK3β is a direct target of miR-101 **A.** The predicted binding sites of miR-101 in the 3′-UTR of GSK3β were conserved from various species. **B.** Luciferase activity of the reporter construct containing the wild-type or mutant 3′UTR of GSK-3β was measured after co-transfection with 0.5 μg miR-101 (left panel) or anti-miR-101 (right panel) in A172 cells. *, *p*<0.05. **C.** The mRNA and protein expression of GSK3β in A172 cells after over-expression of miRNA-101. *, *p*<0.05. **D.** The mRNA and protein expression of GSK3β in A172 cells after transfection with anti-miR-101. *, *p*<0.05. **E.** The inverse correlation between the expression of miR-101 mRNA and GSK3β mRNA in GBM samples (Spearman r: −0.307, p=0.01). **F.** The inverse correlation between miR-101 and GSK3β was confirmed by IHC analysis.

To identify potential target genes of miR-101, we performed *in silico* analysis by using the publicly available databases miRanda, DIANA-microT and TargetScan (Supplementary Figure S2). GSK3β, the gene involved in the regulation of protein synthesis, glycogen metabolism, cell proliferation and survival, was predicted to be one of the potential target genes of miR-101. Besides of GSK3β, other genes such as VEGF and COX-2 have been reported to be targets of miR-101 [19, 20]. However, due to the critical role of GSK3β in TMZ resistance in GBM [21, 22], we chose it for the following studies. As shown in Figure 3A, the predicted binding sites of miR-101 in the 3′-UTR of GSK3β were conserved from various species. To determine whether GSK3β is a direct target gene of miR-101, luciferase reporters fused to wild-type or mutant 3′-UTRs of GSK3β were constructed. Luciferase reporter assay demonstrated that exogenous miR-101 repressed the luciferase activity of wild-type 3′-UTR of GSK3β, but not the luciferase activity of mutant 3′-UTR of GSK3β. By contrast, inhibition of miR-101 by transfection of A172 cells with anti-miR-101 oligonucleotides increased the activity of luciferase reporter fused to the wild-type 3′-UTRs of GSK3β but not the mutant (*, p<0.05; Figure 3B). Furthermore, forced expression of miR-101 in A172 cells reduced GSK3β expression at both the mRNA and protein levels (Figure 3C), whereas suppression of miR-101 enhanced the expression of GSK3β (Figure 3D). The relative expression of miR-101 was shown in Supplementary Figure S1. To confirm the above findings, we analyzed the correlation between miR-101 and GSK3β in GBM samples (n=70). The expression of miR-101 and GSK3β was determined by qRT-PCR. The expression of miR-101 was found to be inversely correlated with GSK3β in the GBM samples (Spearman r: −0.307, p=0.01; Figure 3E). The immunochemistry assay also showed that GBM samples with lower level expression of miR-101 exhibited obvious higher expression of GSK3β (*, p<0.05; Figure 3F). Collectively, these results indicated that GSK3β was a direct target of miR-101 in glioblastoma cells.

### MicroR-101 sensitizes resistant GBM cells to TMZ through downregulation of GSK3β

**Figure 4:**
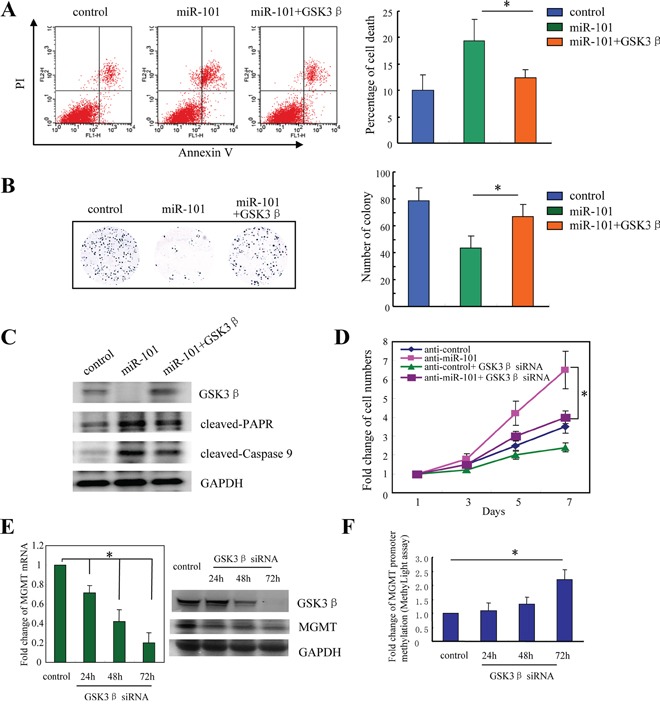
MicroR-101 sensitizes resistant GBM cells to TMZ through downregulation of GSK3β **A, B.** Upon treatment with TMZ (200 μM), A172-TR cells transduced with miR-101 were forced to over-express GSK3β, and then apoptotic analysis (A) and colony formation assay (B) were performed. *, *p*<0.05. **C.** The subsequent apoptotic proteins changes in each group. **D.** A172 cells were transduced with anti-miR-101 or scrambled antisense miRNA control (anti-control), and treated with TMZ (200 μM) for 7 days. Cell numbers were determined every two days. *, *p*<0.05. **E.** The mRNA and protein changes of MGMT expression after knockdown of endogenous GSK3β in T98G cells. *, *p*<0.05. **F.** The effect of GSK3β inhibition on methylation status of the MGMT promoter examined with MethyLight assay in T98G cells. *, *p*<0.05. All data represent the means ± SEM of three replications.

To further evaluate the functional role of GSK3β in miR-101-mediated chemosensitization, we over-expressed GSK3β in TMZ-resistant U251-TR cells transduced with miR-101, and treated those cells with TMZ. The flow cytometry assay showed that U251-TR cells transduced with miR-101 exhibited increased cell apoptosis compared with vector control; however, the increased cell apoptosis caused by miR-101 was attenuated significantly by ectopic over-expression of GSK3β (*, p<0.05; Figure 4A). A similar result was also got from the colony formation assay. miR-101 sensitized resistant U251-TR cells to TMZ treatment, as demonstrated by the decreased colony number formation after ectopic expression of miR-101, whereas GSK3β rescued miR-101-induced cell growth inhibition (*, p<0.05; Figure 4B and Supplementary Figure S3C). These findings were further confirmed by the subsequent protein changes shown in Figure 4C. After exposure to TMZ, expression of both cleaved-PARP and cleaved-Caspase 9 were increased in cells transduced with miR-101, and significantly decreased after forced over-expression of GSK3β (Figure 4C). Furthermore, we knocked down the endogenous GSK3β expression in A172 cells transduced with anti-miR-101 or scrambled antisense miRNA control (anti-control), and treated those cells with TMZ. Cell growth assay showed that suppression of miR-101 rendered A172 cells resistant to TMZ treatment, whereas knockdown of endogenous GSK3β remarkably sensitized these cells to TMZ (*, p<0.05; Figure 4D).

It has been reported that GSK3β inhibition could enhance TMZ effect by silencing O^6^-methylguanine DNA methyltransferase (MGMT) expression via promoter methylation [22]. Therefore, we evaluated whether inhibition of GSK3β could affect MGMT expression in this study. We first examined the changes of MGMT expression after knockdown of endogenous GSK3β in GBM cells. As shown in Figure 4E, both the mRNA and protein expression of MGMT were repressed after inhibition of GSK3β (*, p<0.05). To test the effect of GSK3β inhibition on the methylation of MGMT promoter, we performed a MethyLight assay. The results indicated that GSK3β inhibition significantly increased MGMT promoter methylation (*, p<0.05; Figure 4F). Collectively, these results suggested that miR-101 sensitized resistant GBM cells to TMZ through downregulation of GSK3β.

### Downregulation of miR-101 in GBM patients predicts worse prognosis

**Figure 5:**
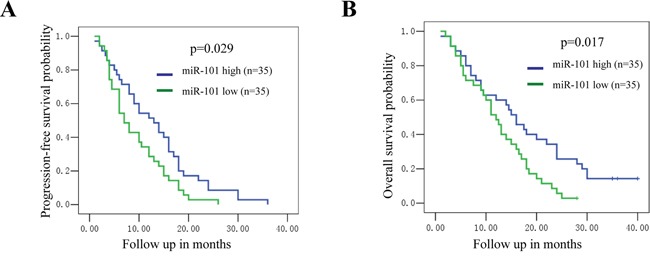
Downregulation of miR-101 in GBM patients predicts worse prognosis **A, B.** Patients with lower expression of miR-101 had both shorter progression-free survival (A; p=0.029) and overall survival (B; p=0.017) as compared to those with higher expression of miR-101.

We investigated the relationship between the expression of miR-101 and clinical characteristics in patients with GBM. As shown in Table 1, no association of miR-101 expression with age, gender, Karnofsky performance score (KPS), tumor size and treatment (resection, chemotherapy and radiotherapy) was found in patients with GBM. However, remarkable correlation of miR-101 expression with differentiation degree of tumor (p=0.017) and disease recurrence (p=0.008) was demonstrated (Table 1). To determine the prognostic value of miR-101 for GBM, Kaplan-Meier survival analysis of patients with GBM was conducted, and log-rank test were performed to evaluate the statistical significance between stratified groups according to the expression of miR-101. MicroR-101 high expression group (miR-101 high) was defined as patients with higher expression level of miR-101 than average and vice versa. As shown in the Figure 5A, patients with lower expression of miR-101 had a significantly shorter progression-free survival (p=0.029). Moreover, patients with lower expression of miR-101 showed a shorter overall survival as compared with those with higher expression of miR-101 (p=0.017). Taken together, these results suggested that downregulation of miR-101 predicted worse prognosis in GBM patients.

**Table 1:** Clinicopathological characteristics of patients with glioma according to the expression of miR-101

Characteristics	miR-101 expression		p value
	Low(n=35)	High(n=35)	
Age (years)			0.334
<45	13	17	
≥45	22	18	
Gender			0.231
Female	14	19	
Male	21	16	
KPS			0.809
<90	19	20	
≥90	16	15	
Tumor size			0.473
<5cm	17	20	
≥5cm	18	15	
Differentiation degree			0.017
Low	22	12	
High	13	23	
Chemotherapy			0.629
Yes	21	19	
No	14	16	
Radiotherapy			0.445
Yes	25	22	
No	10	13	
Resection			0.571
Partial	7	5	
Subtotal	10	14	
Total	18	16	
Recurrence			0.008
Yes	25	14	
No	10	21	

Abbreviation: KPS, Karnofsky performance score.

## DISCUSSION

TMZ is the current first-line chemotherapeutic drug in the treatment of GBM. Resistance to TMZ is a major challenge in GBM therapy [7, 9, 23, 24]. Here, we describe that miRNA-101 may participate in the regulation of TMZ resistance in GBM. In this paper, we show that miR-101 is downregulated in TMZ-resistant GBM cells and human specimens, which is consistent with previous reports in other cancers [25–27]. We also find that miR-101 could sensitize resistant GBM cells to TMZ through downregulation of GSK3β. Importantly, decreased expression of miR-101 is related to poor prognosis in patients with GBM, suggesting that miR-101 can be used as a prognostic molecular marker.

MicroR-101 is a tumor suppressor dysregulated in various malignancies including liver cancer, pancreatic cancer, gastric cancer, lung cancer and ovarian carcinoma, etc [25, 28–34]. In our study, we also found that miR-101 alone could suppress cell growth of GBM. Various studies have shown that downregulation of miRNA-101 is involved in chemoresistance of several cancers [35–37]. It has been reported that downregulation of miR-101 contributes to cisplatin resistance of non-small cell lung cancer cells by targeting ROCK2 [36], miR-101 sensitizes liver cancer cells to doxorubicin-induced cell death through targeting Mcl-1 [38], and miR-101 can enhance chemosensitivity in epithelial ovarian cancer [34]. We confirm that miR-101 also plays a key role in the TMZ resistance of GBM.

While specific miRNAs are well known to be dysregulated in many cancers, correction of these miRNAs using miRNA mimics or antagomiRs could normalize the gene regulatory network and reverse chemoresistance to anti-cancer drugs [39, 40]. MicroRNA based therapeutics appears to be a promising candidate for the development of novel anti-cancer agents, and have been tested in both preclinical and clinical settings [11, 41–43]. Our study has revealed that miR-101 is downregulated and involved in chemoresistance of GBM, it is therefore reasonable to infer that restoration of miR-101 expression may reverse chemoresistance to anti-cancer drugs. Interestingly, our study proves that over-expression of miR-101 does sensitize resistant GBM tumor xenografts to TMZ treatment *in vivo*.

GSK3β is a serine/threonine protein kinase involved in the regulation of protein synthesis, glycogen metabolism, cell proliferation and survival [44, 45]. It is also an important component of diverse signaling pathways including Wnt/β-catenin, insulin, Notch, and Hedgehog signaling pathways [46]. Studies show that it plays a critical role in the development and progression of various malignancies. It has been demonstrated that targeting GSK3β may represent a novel strategy for the treatment of chemoresistant cancers [44]. For instance, downregulation of GSK3β could re-sensitize drug-resistant cells to chemotherapy [47], enhanced expression of GSK3 is associated with acquired resistance to paclitaxel in ovarian cancer [48, 49], and GSK3β inhibition can sensitize human glioblastoma cells to TMZ [22]. In our study, GSK3β is found to be a direct target of miR-101, and miR-101 could sensitize resistant GBM cells to TMZ through downregulation of GSK3β. Moreover, we find that GSK3β inhibition could enhance TMZ effect through repression of MGMT via promoter methylation, which is consistent with previous study that GSK3β could affect MGMT promoter methylation status via c-Myc signaling [22]. It's important to note that GSK3β plays a dual role in GBM either as a tumor suppressor and tumor promoter. For example, Miyashita *et al* showed that GSK3β could promote the survival and proliferation of GBM cells and inhibit apoptosis through p53 and/or Rb-mediated pathways [21]. Chikano *et al* reported that GSK3β enhances invasion of GBM via the focal adhesion kinase, Rac1, and JNK pathway [50]. A recent study revealed that GSK3β could inhibit glioma progression via regulation of mTOR/p70S6K1 signaling pathway [51]. This discrepancy may result from at least in part depends on the signaling environment. The dysregulated downstream effector of GSK3β could have a considerable impact on cell fate and function.

In summary, we have identified that miR-101 is downregulated in TMZ-resistant GBM, rendering GBM cells resistant to TMZ treatment. On the other hand, over-expression of miR-101 can sensitize resistant GBM tumor xenografts to TMZ *in vivo*. It not only yields a better understanding of the underlying mechanisms of miR-101 in TMZ-resistant in GBM, but also paves a new way for novel and powerful anticancer therapeutics. Alternatively, downregulation of miR-101 in GBM patients predicts worse prognosis, suggesting its potential use as a new prognostic marker in GBM.

## MATERIALS AND METHODS

### Tissue samples

The primary and recurrent TMZ-refractory GBM samples were obtained at the First Affiliated Hospital of Zhengzhou University from 2011 to 2016. Tissues were flash frozen immediately after surgery. These samples were collected at the time of diagnosis. All data including age, gender, Karnofsky performance score, tumor size, differentiation degree and locations were obtained from original pathology reports. The study was approved by the Research Ethics Committee of Zhengzhou University, and written informed consent was obtained from all participants.

### Cell culture and transfections

A172, T98G and U-251MG cells were purchased from Cell bank of Chinese Academy of Sciences (Shanghai, China). Cells were cultured in Dulbecco's modified Eagle's medium (Hyclone, Logan, UT, USA) supplemented with 10% fetal bovine serum (Hyclone, Logan, UT, USA), 0.1 mg/ml streptomycin, and 100 units/ml penicillin (Invitrogen, California, USA) in 5% CO_2_ atmosphere at 37°C. Transfection was performed using Lipofectamine 2000 reagent (Invitrogen, Carlsbad, CA, USA) according to the manufacturer's instructions as previously described. The miR-101 mimics, anti-miR-101, GSK3βsiRNA, and their scramble controls were synthesized by Sangon (Shanghai, China).

### Establishment of TMZ-resistant cell lines

The TMZ-resistant cell lines were established as previously reported [52]. Briefly, A172 and U251 cells were exposed to a low dose of TMZ in culture media for 6 months and established TMZ -resistant cells designated as A172-TR and U251-TR, respectively. IC50 value was altered at least 7 to 10 folds increase in TMZ-resistant cells. TMZ was purchased from Sigma (St. Louis, MO) and dissolved in DMSO.

### Lentivirus tranduction

Lentiviral production, titration, and infection were performed as previously described [53]. Briefly, lentiviral plasmids pLV-THM expressing miR-101 or vector control was cotransfected with psPAX2 and pMD2.G plasmids in 293T cells. Lentiviral particles were harvested from the media after 48 hours of transfection, and purified with ultracentrifugation. Cells were then infected with lentiviruses encoding miR-101 or vector control and prepared for the further experiments. Over-expression of miR-101 was confirmed by real-time qPCR analysis.

### RNA extraction and real-time PCR

Total RNA was isolated using the RNeasy mini kit according to the manufacturer's instructions (Qiagen, Germany). The quantity and purity of RNA was quantified by NanoDrop 1000 spectrophotometer (Thermo Scientific, Wilmington, DE, USA). cDNA was prepared using the SuperScript® III First-Strand Synthesis System according to the manufacturer's instructions (Invitrogen, California, USA). Quantitative PCR was performed using SYBR Green dye on an Applied Biosystems 7300 Real-time PCR system (Applied Biosystems, Foster City, CA). The relative gene expression was determined by 2^−ΔΔCT^ method. *GAPDH* and U6 were used as internal control for mRNA and miRNA, respectively.

### Luciferase reporter assays

Luciferase reporter assays were performed as previously reported [54]. Cells were co-transfected with synthetic miR-101 or anti-miR-101, the wild-type or mutant 3′UTR of GSK3β luciferase reporter vector pGL3-GSK3-3′UTR and pRL vector coding for the Renilla luciferase (Promega, Madison, WI, USA). 48 to 72 hours later, cells were collected and luciferase activities were measured using the Dual Luciferase Reporter Assay System (Promega, Madison, WI, USA) according to the manufacturer's instructions.

### Cell proliferation assay

Cell proliferation was determined by Cell Counting Kit-8 assay (Dojindo Laboratories, Kumamoto, Japan) according to the manufacturer's instructions. Cells were plated in 96-well plates and incubated for different periods of time and then added 10 μL of CCK-8 in each well. After incubation for 2 h at 37°C, the absorbance at 450 nm was read on an ELISA plate reader (Model 680, Bio-Rad, CA).

### Western blotting

Western blot analysis was performed as previously described [54]. Briefly, cells were lysed in cold RIPA buffer, proteins (20μg) were resolved on SDS-PAGE, transferred onto PVDF membranes, and probed with antibodies for GSK3β (sc-9166, Santa Cruz Biotechnology), cleaved-PAPR (#9541, Cell Signaling Technology), cleaved-Caspase 9 (#9501, Cell Signaling Technology), MGMT (sc-48805, Santa Cruz Biotechnology), and GAPDH (sc-32233, Santa Cruz Biotechnology) at 4°C overnight. Detection was performed with an enhanced chemiluminescence kit (Santa Cruz, Dallas, TX, USA). The band images were digitally captured and quantified with an Image Lab Software (Bio-Rad, Hercules, CA, USA).

### Immunochemistry

The paraformalin-fixed, paraffin-embedded GBM sections were immunostained using the Dako EnVision™ Flex+ System (K8012; Dako, Glostrup, Denmark). Deparaffinization and epitope unmasking were carried out in a PT-Link using an EnVision™ Flex target retrieval solution (Dako, Carpinteria, CA, USA). The sections were treated with 0.3% hydrogen peroxide (H_2_O_2_) for 5 min to block endogenous peroxidase. Sections were incubated overnight at 4°C with GSK3β (1:100; sc-9166, Santa Cruz Biotechnology). The specimens were subsequently treated with EnVision™ Flex linker rabbit (15 min), EnVision™ Flex/HRP enzyme (30 min), and 3′3-diaminobenzidine tetrahydrochloride (10 min). The samples were counterstained with hematoxylin, dehydrated and mounted on a Richard-Allan Scientific Cyto seal XYL (Thermo Scientific, Waltham, MA, USA). The protein expression was assessed using a combination of the intensity and of percentage positively stained tumor cells to generate a histological score (H-score). The H-score was calculated using the following equation: H-score =ΣPi (i + 1), where i is the intensity score (which ranged 0 ~ 3), and Pi is the percentage of stained tumor cells at each intensity (0% ~ 100%). This formula produces a score that ranges 100 ~ 400, where 100 indicate that 100% of tumor cells were negative and 400 indicates that 100% of tumor cells were strongly stained. The median H-score of GSK3β was used as the cut-off to divide the study cohort into high expression and low expression groups.

### Bioinformatics analysis and target prediction

To predict the binding site of miR-101 and GSK3β, prediction algorithms, including TargetScan (http://www.targetscan.org/vert_71/), DIANA-microT (http://diana.imis.athena-innovation.gr/DianaTools/index.php? r=microT_CDS/index) and microRNA.org (http://www.microrna.org/microrna/home.do) were used for bioinformatics analysis.

### Colony formation assay

1.0 × 10^3^ cells were seeded into 6-well plates (in triplicates) in 2 ml of complete growth medium. The medium of each well was changed every three days. Two to three weeks later, cells were stained by 0.1% crystal violet (Sigma-Aldrich, St. Louis, MO, USA) in methanol for 10 min. The colonies more than 50μm were counted directly on the plate. Statistical significance was calculated from at least three independent experiments.

### MethyLight assay

Genomic DNA was extracted using a QIAamp DNA Mini Kit and subjected to bisulfite conversion using an EpiTect Bisulfite Kit according to the manufacturer's instructions (Qiagen, Germany). MethyLight analysis was performed using the primers and fluorescent probes specific for bisulfite-converted DNA. The primers and probe specific to methylated fraction of the MGMT promoter were taken from previous study [22]. (Probe, 6FAM-CCTTACCTCTAAATACCAACCCCAAACCCG-BHQ-1; forward primer, 5′-CTAACGTATAACGAAAATCGTAACAACC-3′; reverse primer, 5′-AGTATGGAAGGGTAGGAAGAATTCG-3′). Alu was used as reference (probe, 6FAM-CCTACCTTAACCTCCC-BHQ-1; forward primer, 5′-GGTTAGGTATAGTGGTTTATATTTGTAATTTTAGTA-3′; reverse primer, 5′-ATTAACTAAACTAATCTTAAACTCCTAACCTCA-3′). The samples were amplified by using the Epitect MethyLight Mastermix (Qiagen, Germany) according to the manufacturer's protocol in Applied Biosystems 7300 Real-time PCR system (Applied Biosystems, Foster City, CA). Threshold cycle values (Ct) were determined and relative methylation of the MGMT promoter was calculated using the ΔCT method using Alu as a reference gene.

### Tumor xenograft models

Mouse xenograft model were performed as described previously [55]. Briefly, Six to eight weeks old athymic BALB/c nude mice were purchased from Beijing Vital River Laboratory Animal Technology (Beijing, China). The mice were randomly distributed into two groups. TMZ-resistant U251-TR cells stably transduced with either miR-101 or vector control were injected subcutaneously into two groups of nude mice (n = 5). The mice were injected intraperitoneally with TMZ (20 mg/kg/day) every three days after two weeks of implantation. The tumor size was measured with calipers every three days, and tumor volume was calculated using the formula (Length × Width^2^/2). Five to six weeks after implantation, the xenograft tumor began to appear at the site of implantation with 0.5 to 1.0 cm^3^ in volume. Mice were euthanized by asphyxiation in a CO_2_ chamber and tumors were excised using standard procedure. All animal experiments were conducted with the approval of the Animal Care and Use Committee of Zhengzhou University.

### Statistical analysis

All data were expressed as mean ±standard error of the mean (SEM). Between groups and among groups comparisons were conducted with Student t test and ANOVA, respectively. Mann-Whitney U test was used for nonparametric variables. The association of miR-101 expression and clinicopathological characteristics was analyzed by Chi-square or Fisher's two-tailed exact test. Progress-free survival (PFS) and overall survival (OS) rates were calculated using the Kaplan–Meier method and the significance was assessed by the log-rank test. Statistical analysis was performed using GraphPad Prism software version 4.0 (PRISM4) (GraphPad Software Inc, LaJolla, CA), and *p*<0.05 was considered significant.

## SUPPLEMENTARY FIGURES


