# Supplementary material for: MicroRNA-101 reverses temozolomide resistance by inhibition of GSK3β in glioblastoma
Source: Oncotarget. 2016 Oct 25;7(48):79584–95. doi: 10.18632/oncotarget.12861 (PMC5346737; doi:10.18632/oncotarget.12861)
Supplement: Supplementary file 1 [file oncotarget-07-79584-s001.pdf]

MicroRNA-101 reverses temozolomide resistance by inhibition of GSK3β in glioblastoma

SUPPLEMENTARY FIGURES

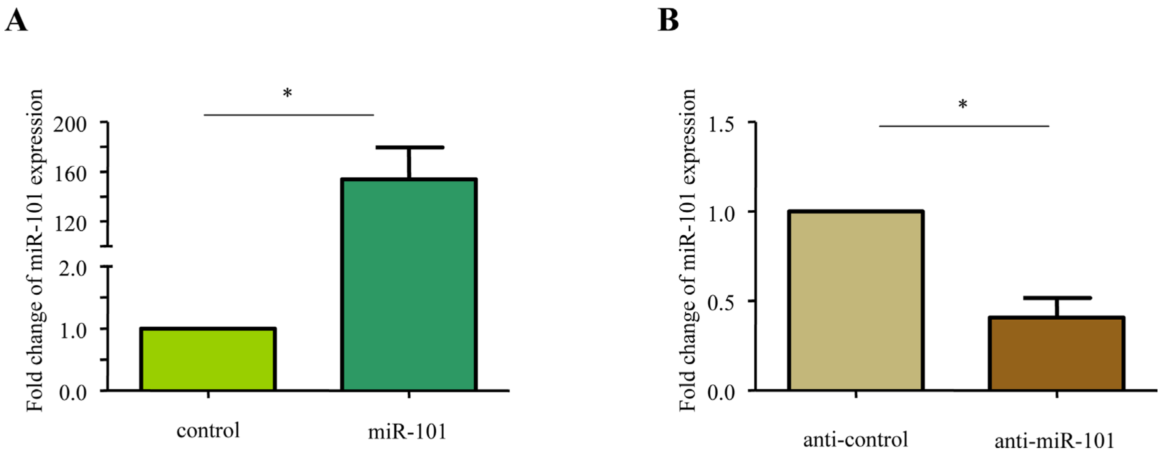

Supplementary Figure S1: The relative expression of miR-101 in A172 cells either over-expression of miRNA-101 A. or downregulation by anti-miR-101 **B.**

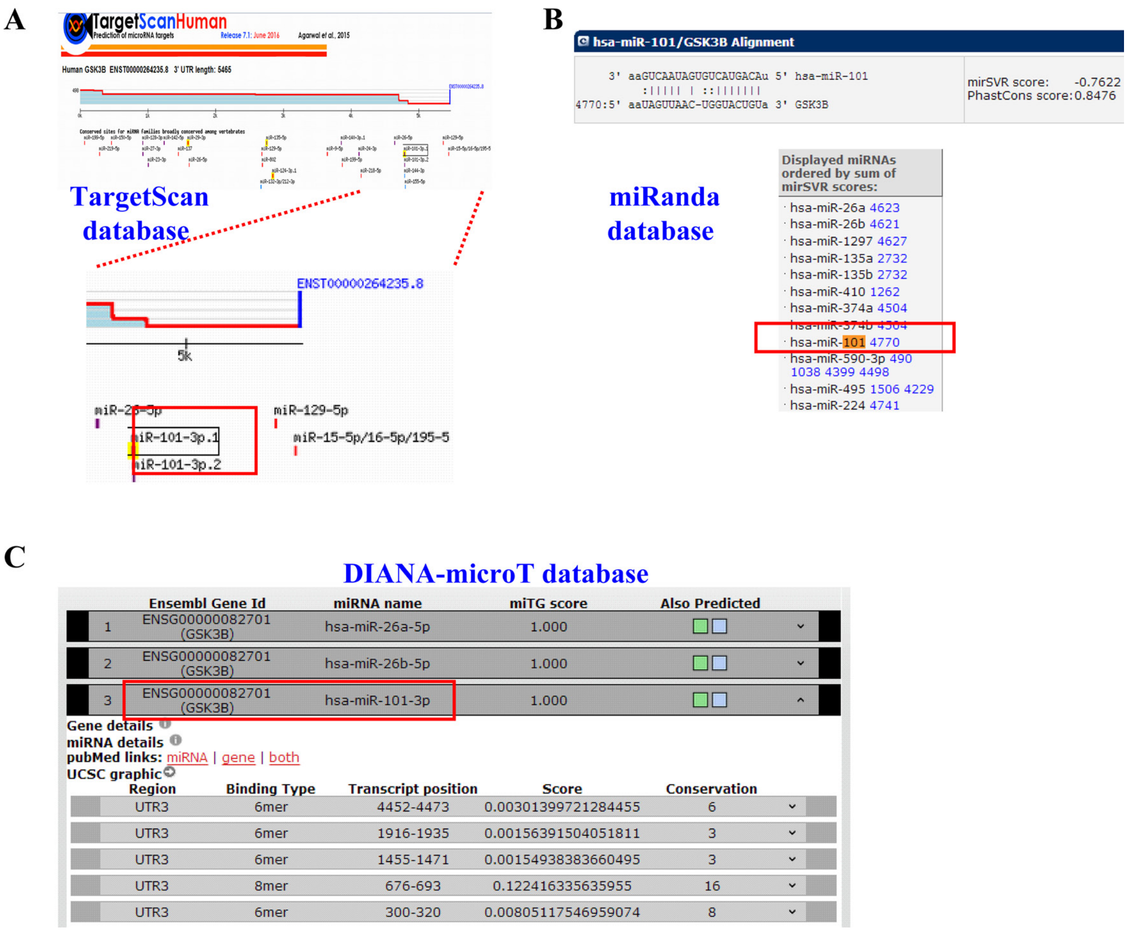

Supplementary Figure S2: In silico analysis used by the publicly available databases TargetScan A. miRanda **B.** and DIANA-microT **C.** showed that GSK3β was the potential target gene of miR-101**.**

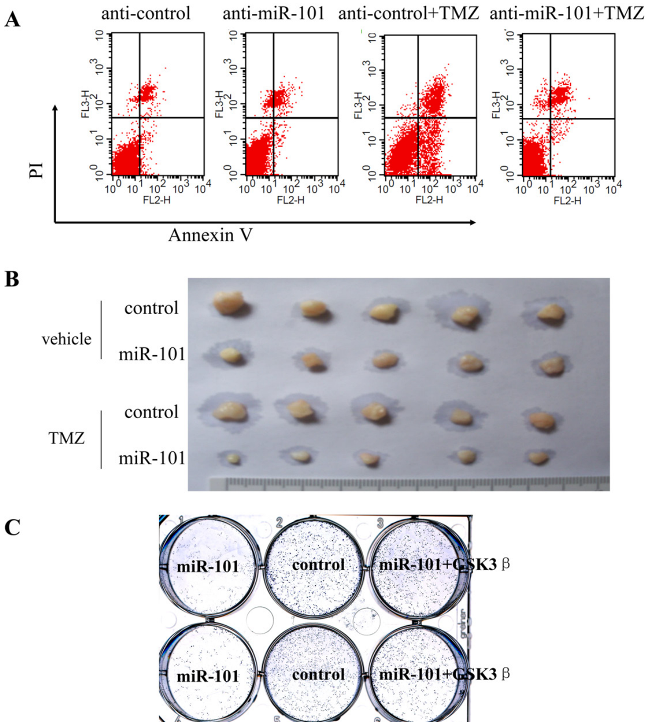

Supplementary Figure S3: A. The percentage of cell death of A172 cells transfected with anti-miR-101 or control in the presence or absence of TMZ. **B.** Tumor xenografts generated in mice four weeks after inoculation. **C.** The colony formation assay of A172-TR cells transduced with miR-101 and GSK3β upon the treatment of TMZ (200 μM).
